# Editorial: Diabetic kidney disease: routes to drug development, pharmacology and underlying molecular mechanisms

**DOI:** 10.3389/fphar.2023.1252315

**Published:** 2023-08-08

**Authors:** Divya Bhatia, Swayam Prakash Srivastava

**Affiliations:** ^1^ Division of Nephrology and Hypertension, Joan and Sanford I. Weill Department of Medicine, New York, NY, United States; ^2^ NewYork-Presbyterian Hospital, Weill Cornell Medicine, New York, NY, United States; ^3^ Division of Regenerative Medicine, Department of Medicine, Hartman Institute of Therapeutic Organ Regeneration, Weill Cornell Medicine, New York, NY, United States

**Keywords:** diabetic kidney disease, sodium-glucose cotransporter-2 (SGLT2) inhibitors, dipeptidyl peptidase-4 (DPP4 or CD26) inhibitors, N-acetyl-seryl-aspartyl-lysyl-proline (Ac-SDKP), sirtuin (SIRT)3, mitoquinone (MitoQ), kidney fibrosis, mitophagy

## Introduction

Diabetic kidney disease (DKD) is one of the leading causes of kidney failure, which leads to end-stage renal disease (ESRD) and affects almost one-third of the total diabetic patients worldwide (Cooper and Warren, 2019; Tuttle et al., 2022). DKD is also one of the significant long-term complications, which not only imposes healthcare costs on patients with diabetes but substantially increases the rates of morbidity and mortality. A wide knowledge gap exists between biology and drug development which contributes to the suboptimal treatment options against DKD. An increased understanding of DKD is urgently needed for the development of novel therapeutics, which will help in targeting the changes that occur during the early stages of the disease. Several attempts have also been made to hybridize the process of biomarker discovery and drug development to arrest and reverse the inevitable events, which occur during the progression of DKD. However, currently approved therapeutic regimens such as angiotensin-converting enzyme (ACE) inhibitors, angiotensin II receptor blockers (ARBs), and statins, etc. can simply delay, but do not prevent the decline in kidney function and progression of ESRD (Gentile et al., 2014; Srivastava et al., 2020a; Hartman et al., 2020). Although these therapeutic agents help in reducing albuminuria in patients with DKD, they expose patients to multiple adverse reactions and drug intolerance. Thus, to improve kidney function in diabetic patients, there is an unmet need for new therapeutic strategies.

A variety of pharmacological agents are currently in clinical trials such as sodium glucose transporter-2 (SGLT-2) inhibitors and in preclinical settings including dipeptidase transferase-4 (DPP-4) inhibitors, mineralocorticoid receptor antagonists, N-acetyl-seryl-aspartyl-lysyl-proline (Ac-SDKP), and endothelin receptor antagonists (ERAs) (Kanasaki et al., 2014; Li et al., 2020b; Srivastava et al., 2020b). These molecules have been shown to exhibit protective effects against increases in inflammatory and fibrotic responses in the kidney and worsening of kidney function in preclinical settings.

In this Research Topic, we provided a deeper insight into the recent advances in the pathophysiologic pathways, which can be modulated to prevent the worsening of diverse phenotypes of DKD. We also addressed the molecular mediators, which can be targeted to attenuate the progression of kidney fibrosis during DKD. Understanding these critical pathways and potential mediators will guide the discovery process of future therapeutic approaches against DKD.

Broadly we categorized this editorial into two major sections.

## New molecular mechanisms in DKD

In recent years, we have worked on identifying new cellular and molecular mechanisms causative or protective in DKD. Defective central metabolism, i.e., reduced fatty acid oxidation (FAO) and induced abnormal glycolysis, accelerates the mesenchymal switching, and finally fibrogenesis (Kang et al., 2015; Srivastava et al., 2018). Dichloroacetate, 2-deoxyglucose, and C75 by correcting defective metabolism via promoting FAO levels in tubules, help in abolishing the fibrotic phenotype and improving kidney structure and functions (Kang et al., 2015). Sirtuin (SIRT)3 is the key regulator of central metabolism. SIRT3 induction provides protection to both tubules and kidney vasculature. SIRT3 abrogates abnormal glycolysis and improves lipid metabolism by transconversion of pyruvate kinase isozymes M1/M2 (PKM2) dimer to tetramer. SIRT3 also reduces the accumulation of hypoxia-inducible factor (HIF)-1α in the kidney tubules and endothelium (Srivastava et al., 2018; Srivastava et al., 2021b).

In this Research Topic, Li et al. described that Yin and Yang (YY)1 is involved in the upregulation LncRNA-ARAP1-AS2, which by promoting aberrant glycolysis caused fibrosis. The impaired glycolysis was associated with the activation of epidermal growth factor receptor (EGFR)/PKM2/HIF1α pathway. Their results highlight the role of YY1 in promoting glycolysis during DKD and suggest it as a potential therapeutic strategy for consideration.

Other molecules, i.e., endothelium glucocorticoid receptors (GR) and podocyte glucocorticoid receptors are critical in restoring cellular antifibrotic mechanisms, abolishing the mesenchymal metabolic shift and, associated kidney inflammation and fibrosis in diabetes (Srivastava et al., 2021c; Srivastava et al., 2021d). Glucocorticoid receptor negatively targets canonical Wnt signaling pathway, inflammation, and improves tubular and endothelial health (Srivastava et al., 2021d). Kidney endothelium-specific deficiency of SIRT3, GR, or fibroblast growth factor receptor (FGFR)1 accelerates the endothelial-to-mesenchymal transition (EndMT) by disrupting neighboring cell homeostasis and promoting epithelial–mesenchymal transition (EMT) in tubular cells during diabetes (Li et al., 2020a; Srivastava et al., 2021b; Srivastava et al., 2021d). This EndMT-mediated activation of partial EMT is caused due to systemic inflammation, accompanied by elevating levels of pro-mesenchymal and fibrotic cytokines, and reducing levels of anti-mesenchymal cytokines. Cumulative effects of systemic inflammation, defective central metabolism, and upregulated mesenchymal transcription factors, finally contribute to severe fibrosis in diabetic kidneys. Another article in this Research Topic described the role of nucleotide-binding oligomerization domain (NOD)-like receptors (NLPR3)-mediated pyroptosis in diabetic nephropathy (Wan et al.). This study demonstrated that the canonical NLRP3 inflammasome pathway, which involves the formation of membrane pores, and promotes inflammation, can also induce pyroptosis in the kidneys during DKD. This study suggests that targeting pyroptosis-associated proteins has therapeutic potential in the management of diabetes and related kidney disease.

Another study in this Research Topic by Cen et al. reported the protective effects of tetramethylpyrazine nitrone (TBN) in improving renal anemia, by maintaining iron homeostasis through activating the HIF via 5′ adenosine monophosphate-activated protein kinase (AMPK)/mammalian target of rapamycin (mTOR)/4E-binding protein 1 (4E-BP1) pathway. TBN, a nitrone derivative of tetramethylpyrazine also acts as a free radical scavenger against hydroxyl, superoxide, and peroxynitrite. Recently, we reported the therapeutic benefits of repleting the labile iron pool (LIP) of kidney macrophages in improving their antioxidant response and attenuating inflammation, and fibrosis during chronic kidney disease (CKD) (Patino et al., 2023). We also observed that the expression of superoxide dismutase-2 (SOD-2), an antioxidant enzyme decreased in the kidney during CKD, which was associated with an increase in mROS production by kidney macrophages (Bhatia et al., 2022). Therefore, it is critical to further investigate the therapeutic benefits of TBN as reported by Cen et al. against increases in oxidative stress, inflammation, and CKD-associated anemia.

## New molecules against DKD

The discovery of SGLT-2 inhibitors has influential effects in the treatment of diabetes and associated kidney disease. The data from preclinical settings suggest that SGLT-2 inhibitors not only reduced worsening of kidney function and fibrosis, but, improved the overall structural impairments in the kidney of patients with DKD. The data from EMPA-REG OUTCOME clinical trials demonstrated that the use of SGLT-2 inhibitor, empagliflozin is safe and effective in reducing renal-related complications in patients with type 2 diabetes, suggesting the remarkable discovery in the treatment of DKD (Mayer et al., 2019). The SGLT-2 inhibitors provide protection against DKD through promoting the excretion of blood glucose in the urine and reducing intraglomerular pressure. However, the data from the diabetic mouse models have shown that the empagliflozin did not cause any change in blood pressure and blood glucose levels. These data from experimental models suggested that empagliflozin was able to reduce the aberrant glycolysis and induce the FAO in the tubules (Li et al., 2020b). Increased aberrant glycolysis and diminished FAO promote mesenchymal metabolic shift, which contributes to the deterioration of kidney function, structural abnormalities, and kidney fibrosis (Srivastava et al., 2018).

In this Research Topic, Hu et al. performed a systematic review to understand the efficacy and safety of SGLT-2 inhibitors in patients stratified in different groups based on their estimated glomerular filtration rate (eGFR). Based on the search using four databases, they reported that the SGLT2 inhibitors decreased the glycated hemoglobin (HbA1c) levels and body weight, which were in parallel with the baseline eGFR levels. However, SGLT2 inhibitor-mediated reductions in blood pressure levels were independent of the baseline eGFR in patients. Other reported potential therapeutic mediators, which have also shown to be effective against DKD include DPP-4 inhibitors and incretins analogs. Among different classes of DPP-4 inhibitors, linagliptin has been reported to be most effective in combating diabetes and associated kidney disease (Kanasaki et al., 2014). Linagliptin intake increased the levels of Ac-SDKP tetrapeptide and exhibited renal protection by delaying the progression of kidney disease.

Plant-derived synthetic compounds such as ursolic acid, paeoniflorin, and saponins are found to be effective against DKD. Interestingly ursolic acid improved the renal structural impairments and function through elevating FGFR1 and SIRT3 levels and reducing the expression of DPP-4 (Liu et al.). DPP-4 is known to exhibit profibrotic functions in the tubules. FGFR1 activation is related to the upregulation of microRNAs (miRNAs), such as miR-let-7s and miR-29s, these miRNAs exert antifibrotic effects (Srivastava et al., 2016). In addition, induction of SIRT3 also exhibits protective effects by abolishing the mesenchymal metabolism shift. Ac-SDKP mediates renal protection by binding with FGFR1 and elevating SIRT3 levels (Li et al., 2017; Srivastava et al., 2020b). Therefore, this tetrapeptide has great potential as a future therapeutic option for combating diabetes and related kidney disease (Srivastava et al., 2021a). The author Fang et al. reported that the Niaoduqing molecules mitigate high-glucose-induced podocyte injury by regulating advanced glycation endproducts/receptor for AGE (AGE/RAGE) signaling pathway.

The activation of SIRT3 is also known to attenuate NLRP3-mediated inflammation, apoptosis, and kidney fibrosis during DKD by inducing mitophagic activities (Feng et al., 2018). Mitochondrial-targeted approaches have also shown great efficacy against DKD in experimental models and patients (Bhatia et al., 2020). Mitoquinone (MitoQ), a mitochondrial-targeted coenzyme Q has been reported to mitigate albuminuria, glomerular hypertrophy, mesangial matrix expansion, and kidney fibrosis by restoring the expression of mitophagy regulators, including mitofusin (MFN)-2 and reducing mitochondrial fragmentation (Xiao et al., 2017). We have also observed the myeloid lineage-specific protective functions of MFN2 against increases in kidney macrophage-derived inflammatory and fibrotic responses during CKD (Bhatia et al., 2022). The efficacy of MitoQ was also investigated as part of a Phase IV controlled, double-blind clinical trial in stage 3–5 patients with CKD (ClinicalTrials.gov, Identifier number: NCT02364648). Szeto-Schiller-31 peptide (H-D-Arg-Dmt-Lys-Phe-NH2) (SS-31), a mitochondrial antioxidant also reduced macrophage recruitment, glomerulosclerosis, podocyte and endothelial cell death in the experimental model of DKD (Szeto et al., 2016).

In this Research Topic, a study by Wang et al. reported that sappanone A (SA), a plant-derived homoisoflavanone, prevented the worsening of kidney function in uninephrectomized and streptozotocin-induced DKD, by restoring the expression of nuclear factor kappa B (NF-κB) inhibitor alpha (IκBα). Their findings highlighted the protective effects of SA against increases in pro-inflammatory (interleukin-1β and tumor necrosis factor-α) and pro-fibrotic (transforming growth factor-β1 and collagen-IV) responses in the kidney during DKD. Restoration of IκBα is also critical in averting mitochondrial fragmentation and cytochrome (Cyt C)-mediated apoptotic cell death (Laforge et al., 2016). We also observed increases in circulating and urinary levels of Cyt C in experimental and human kidney fibrosis (Bhatia et al., 2022). The increased levels of Cyt C were associated with macrophage-mediated inflammatory and fibrotic events in the kidney (Bhatia et al., 2022). Therefore, the study by Wang et al. is important in confirming, that the SA-dependent protective effects against increases in inflammation and cell death during DKD are exerted via suppression of NF-κB pathway.

Another study by Chen et al. reviewed the therapeutic benefits of Chinese herbal medicines in suppressing oxidative stress, inflammation, and mitochondrial dysfunction. They highlighted that combining these herbal medicines with conventional therapies may further delay the progression of DKD effectively. These herbal medicines have been shown to modulate various pathways, including, i) Glucagon-like Peptide-1 (GLP)-receptor, ii) SGLT2, iii) SIRT1/AMPK, iv) AGE/RAGE, v) NF-κB, vi) nuclear factor erythroid 2–related factor 2 (Nrf2), vii) NLRP3, viii) Peroxisome proliferator-activated receptor-gamma coactivator (PGC)-1α, and ix) PINK1/Parkin-mediated mitophagy. We also reported that PINK1/Parkin-mediated mitophagy is downregulated in experimental and human kidney fibrosis (Bhatia et al., 2019). The expression of PGC-1α, which is a chief regulator of mitochondrial biogenesis has also been found to be suppressed in the kidney during fibrosis (Bhatia et al., 2022). Fenofibrate has been reported to promote mitochondrial fatty acid β-oxidation and regulate kidney function in patients with DKD by activating PGC-1α (Davis et al., 2011). Considering that kidneys are the second richest organ after the heart in mitochondrial content, it is anticipated and widely reported that mitochondrial dysfunction exaggerates the worsening of kidney diseases including DKD (Forbes and Thorburn, 2018; Bhatia and Choi, 2019; Bhatia et al., 2020). Mitophagy helps in the recycling of damaged mitochondrial components, which can act as mitochondrial damaged-associated molecular patterns (mtDAMPs) and promote inflammatory events in the kidney during DKD (Bhatia and Choi, 2023). These mtDAMPs, which include mitochondrial-derived reactive oxygen species (mROS), Cyt C, and cell-free or intracellular naked mitochondrial DNA (mtDNA) can exaggerate inflammation and fibrosis via activating NF-κB by toll-like receptor (TLR)-mediated signaling pathway. The mechanism of action of Chinese herbal medicines against the progression of DKD warrants further investigation.

## Conclusion

Diabetic kidney disease is the chief cause of kidney failure and ESRD, which also increases cardiovascular events and mortality in the suffering patient population globally. It is important to understand the underlying mechanisms of diabetic milieu-induced kidney damage and progression of DKD. In this Research Topic, we have discussed potent therapeutic regimens for the management of DKD, tissue/cell/mitochondrial-specific novel biological mechanistic approaches, functions of regulatory non-coding RNAs, and clinical data sets. We speculate that this Research Topic will provide basic essential information, that could be used in the design of potential therapeutic agents and help in the management of patients with DKD.

## References

[B1] Bhatia D. Choi M. E. (2019). The emerging role of mitophagy in kidney diseases. J. Life Sci. (Westlake Village) 3, 13–22. 10.36069/jols/20191203 PMC7041910 32099974

[B2] Bhatia D. Choi M. E. (2023). Autophagy and mitophagy: physiological implications in kidney inflammation and diseases. Am. J. Physiol. Ren. Physiol. 325, F1–F21. 10.1152/ajprenal.00012.2023 PMC10292977 37167272

[B3] Bhatia D. Chung K. P. Nakahira K. Patino E. Rice M. C. Torres L. K. (2019). Mitophagy-dependent macrophage reprogramming protects against kidney fibrosis. JCI Insight 4, e132826. 10.1172/jci.insight.132826 31639106 PMC6962025

[B4] Bhatia D. Capili A. Choi M. E. (2020). Mitochondrial dysfunction in kidney injury, inflammation, and disease: potential therapeutic approaches. Kidney Res. Clin. Pract. 39, 244–258. 10.23876/j.krcp.20.082 32868492 PMC7530368

[B5] Bhatia D. Capili A. Nakahira K. Muthukumar T. Torres L. K. Choi A. M. K. (2022). Conditional deletion of myeloid-specific mitofusin 2 but not mitofusin 1 promotes kidney fibrosis. Kidney Int. 101, 963–986. 10.1016/j.kint.2022.01.030 35227692 PMC9038692

[B6] Cooper M. Warren A. M. (2019). A promising outlook for diabetic kidney disease. Nat. Rev. Nephrol. 15, 68–70. 10.1038/s41581-018-0092-5 30532022

[B7] Davis T. M. Ting R. Best J. D. Donoghoe M. W. Drury P. L. Sullivan D. R. (2011). Effects of fenofibrate on renal function in patients with type 2 diabetes mellitus: the fenofibrate intervention and event lowering in diabetes (FIELD) study. Diabetologia 54, 280–290. 10.1007/s00125-010-1951-1 21052978

[B8] Feng J. Lu C. Dai Q. Sheng J. Xu M. (2018). SIRT3 facilitates amniotic fluid stem cells to repair diabetic nephropathy through protecting mitochondrial homeostasis by modulation of mitophagy. Cell. Physiol. Biochem. 46, 1508–1524. 10.1159/000489194 29689547

[B9] Forbes J. M. Thorburn D. R. (2018). Mitochondrial dysfunction in diabetic kidney disease. Nat. Rev. Nephrol. 14, 291–312. 10.1038/nrneph.2018.9 29456246

[B10] Gentile G. Mastroluca D. Ruggenenti P. Remuzzi G. (2014). Novel effective drugs for diabetic kidney disease? Or not? Expert Opin. Emerg. Drugs 19, 571–601. 10.1517/14728214.2014.979151 25376947

[B11] Hartman R. E. Rao P. S. S. Churchwell M. D. Lewis S. J. (2020). Novel therapeutic agents for the treatment of diabetic kidney disease. Expert Opin. Investig. Drugs 29, 1277–1293. 10.1080/13543784.2020.1811231 32799584

[B12] Kanasaki K. Shi S. Kanasaki M. He J. Nagai T. Nakamura Y. (2014). Linagliptin-mediated DPP-4 inhibition ameliorates kidney fibrosis in streptozotocin-induced diabetic mice by inhibiting endothelial-to-mesenchymal transition in a therapeutic regimen. Diabetes 63, 2120–2131. 10.2337/db13-1029 24574044

[B13] Kang H. M. Ahn S. H. Choi P. Ko Y. A. Han S. H. Chinga F. (2015). Defective fatty acid oxidation in renal tubular epithelial cells has a key role in kidney fibrosis development. Nat. Med. 21, 37–46. 10.1038/nm.3762 25419705 PMC4444078

[B14] Laforge M. Rodrigues V. Silvestre R. Gautier C. Weil R. Corti O. (2016). NF-κB pathway controls mitochondrial dynamics. Cell. Death Differ. 23, 89–98. 10.1038/cdd.2015.42 26024391 PMC4815975

[B15] Li J. Shi S. Srivastava S. P. Kitada M. Nagai T. Nitta K. (2017). FGFR1 is critical for the anti-endothelial mesenchymal transition effect of N-acetyl-seryl-aspartyl-lysyl-proline via induction of the MAP4K4 pathway. Cell. Death Dis. 8, e2965. 10.1038/cddis.2017.353 28771231 PMC5596544

[B16] Li J. Liu H. Srivastava S. P. Hu Q. Gao R. Li S. (2020a). Endothelial FGFR1 (fibroblast growth factor receptor 1) deficiency contributes differential fibrogenic effects in kidney and heart of diabetic mice. Hypertension 76, 1935–1944. 10.1161/HYPERTENSIONAHA.120.15587 33131311

[B17] Li J. Liu H. Takagi S. Nitta K. Kitada M. Srivastava S. P. (2020b). Renal protective effects of empagliflozin via inhibition of EMT and aberrant glycolysis in proximal tubules. JCI Insight 5, e129034. 10.1172/jci.insight.129034 32134397 PMC7213787

[B18] Mayer G. J. Wanner C. Weir M. R. Inzucchi S. E. Koitka-Weber A. Hantel S. (2019). Analysis from the EMPA-REG OUTCOME^®^ trial indicates empagliflozin may assist in preventing the progression of chronic kidney disease in patients with type 2 diabetes irrespective of medications that alter intrarenal hemodynamics. Kidney Int. 96, 489–504. 10.1016/j.kint.2019.02.033 31142441

[B19] Patino E. Bhatia D. Vance S. Z. Antypiuk A. Uni R. Campbell C. (2023). Iron therapy mitigates chronic kidney disease progression by regulating intracellular iron status of kidney macrophages. JCI Insight 8, e159235. 10.1172/jci.insight.159235 36394951 PMC9870080

[B20] Srivastava S. P. Shi S. Kanasaki M. Nagai T. Kitada M. He J. (2016). Effect of antifibrotic MicroRNAs crosstalk on the action of N-acetyl-seryl-aspartyl-lysyl-proline in diabetes-related kidney fibrosis. Sci. Rep. 6, 29884. 10.1038/srep29884 27425816 PMC4947922

[B21] Srivastava S. P. Li J. Kitada M. Fujita H. Yamada Y. Goodwin J. E. (2018). SIRT3 deficiency leads to induction of abnormal glycolysis in diabetic kidney with fibrosis. Cell. Death Dis. 9, 997. 10.1038/s41419-018-1057-0 30250024 PMC6155322

[B22] Srivastava S. P. Goodwin J. E. Kanasaki K. Koya D. (2020a). Inhibition of angiotensin-converting enzyme ameliorates renal fibrosis by mitigating DPP-4 level and restoring antifibrotic MicroRNAs. Genes. (Basel) 11, 211. 10.3390/genes11020211 32085655 PMC7074526

[B23] Srivastava S. P. Goodwin J. E. Kanasaki K. Koya D. (2020b). Metabolic reprogramming by N-acetyl-seryl-aspartyl-lysyl-proline protects against diabetic kidney disease. Br. J. Pharmacol. 177, 3691–3711. 10.1111/bph.15087 32352559 PMC7393199

[B24] Srivastava S. P. Kanasaki K. Goodwin J. E. (2021a). Editorial: combating diabetes and diabetic kidney disease. Front. Pharmacol. 12, 716029. 10.3389/fphar.2021.716029 34305620 PMC8295890

[B25] Srivastava S. P. Li J. Takagaki Y. Kitada M. Goodwin J. E. Kanasaki K. (2021b). Endothelial SIRT3 regulates myofibroblast metabolic shifts in diabetic kidneys. iScience 24, 102390. 10.1016/j.isci.2021.102390 33981977 PMC8086030

[B26] Srivastava S. P. Zhou H. Setia O. Dardik A. Fernandez-Hernando C. Goodwin J. (2021c). Podocyte glucocorticoid receptors are essential for glomerular endothelial cell homeostasis in diabetes mellitus. J. Am. Heart Assoc. 10, e019437. 10.1161/JAHA.120.019437 34308664 PMC8475689

[B27] Srivastava S. P. Zhou H. Setia O. Liu B. Kanasaki K. Koya D. (2021d). Loss of endothelial glucocorticoid receptor accelerates diabetic nephropathy. Nat. Commun. 12, 2368. 10.1038/s41467-021-22617-y 33888696 PMC8062600

[B28] Szeto H. H. Liu S. Soong Y. Alam N. Prusky G. T. Seshan S. V. (2016). Protection of mitochondria prevents high-fat diet-induced glomerulopathy and proximal tubular injury. Kidney Int. 90, 997–1011. 10.1016/j.kint.2016.06.013 27519664

[B29] Tuttle K. R. Agarwal R. Alpers C. E. Bakris G. L. Brosius F. C. Kolkhof P. (2022). Molecular mechanisms and therapeutic targets for diabetic kidney disease. Kidney Int. 102, 248–260. 10.1016/j.kint.2022.05.012 35661785

[B30] Xiao L. Xu X. Zhang F. Wang M. Xu Y. Tang D. (2017). The mitochondria-targeted antioxidant MitoQ ameliorated tubular injury mediated by mitophagy in diabetic kidney disease via Nrf2/PINK1. Redox Biol. 11, 297–311. 10.1016/j.redox.2016.12.022 28033563 PMC5196243

